# Defective Interferon Gamma Production by Tumor-Specific CD8^+^ T Cells Is Associated With 5′Methylcytosine-Guanine Hypermethylation of Interferon Gamma Promoter

**DOI:** 10.3389/fimmu.2020.00310

**Published:** 2020-03-05

**Authors:** Megat Abd Hamid, Xuan Yao, Craig Waugh, Samara Rosendo-Machado, Chris Li, Timothy Rostron, John Frankland, Yanchun Peng, Tao Dong

**Affiliations:** ^1^Chinese Academy of Medical Sciences (CAMS) Oxford Institute, Nuffield Department of Medicine, University of Oxford, Oxford, United Kingdom; ^2^Medical Research Council (MRC) Human Immunology Unit, MRC Weatherall Institute of Molecular Medicine, Radcliffe Department of Medicine, University of Oxford, Oxford, United Kingdom; ^3^Flow Cytometry Facility, MRC Weatherall Institute of Molecular Medicine, University of Oxford, Oxford, United Kingdom; ^4^Sequencing Facility, MRC Weatherall Institute of Molecular Medicine, University of Oxford, Oxford, United Kingdom

**Keywords:** methylation, mRNA, interferon gamma, promoter, CD8^+^ T cells, response

## Abstract

Interferon gamma (IFNγ) supports effector responses of CD8^+^ cytotoxic T lymphocytes (CTLs) and is a surrogate marker for detection of antigen-specific T cells. Here, we show that tumor-specific CTL clones have impaired IFNγ expression and production upon activation. Assessment of the relationship between IFNγ production and the 5′methylcytosine-guanine (CpG) dinucleotide methylation of the IFNγ promoter using bisulfite treatment has shown that IFNγ^−^ CTL clones accumulates CpG hypermethylation within the promoter at key transcription factor binding sites (−186 and −54), known to be vital for transcription. We confirmed these findings using *ex vivo* isolated and short-term expanded bulk tumor-specific CTL lines from four cancer patients and demonstrated that IFNγ methylation inversely correlates with transcription, protein level, and cytotoxicity. Altogether, we propose that a sizeable portion of human tumor-specific CTLs are deficient in IFNγ response, contributed by CpG hypermethylation of the IFNγ promoter. Our findings have important implications for immunotherapy strategies and for methods to detect human antigen-specific T cells.

## Introduction

Antigen-specific immunity drives the production of interferon gamma (IFNγ; a type II interferon) by CD8^+^ cytotoxic T lymphocytes (CTLs), which helps to control virus infection and cancer (1, 2). IFNγ expression is commonly used as a functional surrogate for CTLs. The current common methods to detect human antigen-specific T cells are often based on IFNγ production upon antigen stimulation, such as enzyme-linked immunospot (ELISpot) assay and intracellular cytokine staining (ICS) assay (3, 4).

IFNγ expression in T cells is driven by T cell receptor (TCR)-mediated downstream signaling, particularly the NF-κB pathway (5). NF-κB signaling phosphorylates and activates several transcription factors, including cAMP response element binding protein (CREB), activating transcription factor (ATF), and activator protein-1 (AP-1). These transcription factors translocate into the nucleus upon activation and bind upstream of the IFNγ promoter to initiate mRNA transcription (6, 7).

Cytosine methylation at position 5 of its pyrimidine ring (5′methylcytosine) is the most abundant DNA modification in the genome. 5′Methylcytosine-guanine (CpG) methylation at gene promoters can stably silence genes by preventing transcription factor accessibility and protein expression (8). Many important genes involved in immunity are regulated by CpG methylation. For instance, the IFNγ promoter is demethylated and expressed in Th1-differentiated CD4^+^ T cells but not in naïve CD4^+^ T cells and Th2-differentiated T cells. It is also likely that methylation can potentially influence the memory CD8^+^ T cell epigenetics (9, 10).

Cancer can employ diverse mechanisms to induce T cell exhaustion and escape anti-tumor T cell immunity, including by downregulating the MHC class Ia expression on cancer cells and by dysregulating inhibitory receptors expression, such as PD-1 on tumor-infiltrating T lymphocytes (TILs) (11–13). In addition, epigenetic mechanisms that dysregulate cytokine expression have been suggested to be another manifestation of T cell exhaustion (14–16). Previous studies have shown that fewer memory and effector CD4^+^ and CD8^+^ T cells from peripheral blood of cancer patients can produce IFNγ in contrast to those of healthy individuals, whereas IFNγ-deficient mice, compared with wild-type mice, have demonstrated reduced migration of activated T cells to tumor sites and enhanced tumor progression (17, 18). This highlighted the importance of IFNγ in mediating anti-tumor T cell responses. However, it still remains unclear if tumor-specific CTLs can have deficient IFNγ responses and the factors contributing to the deficiency.

Different types of cancer express “cancer-testis antigens” (CTAs), a distinct class of antigens that are normally expressed in germ cells of the testis but not in other normal tissues. This family of antigens includes synovial sarcoma X (SSX) and New York-esophageal squamous cell carcinoma-1 (NY-ESO-1) proteins. CTAs are thought to be promising targets for immunotherapy, as they are highly overexpressed in cancer cells and are recognized by CTLs.

Here, we examined IFNγ responses and IFNγ promoter CpG methylation in CTA-specific CTL clones and short-term primary T cell lines. We found that several tumor-specific CD8^+^ T cell clones did not transcribe nor produce IFNγ upon T cell activation, correlating with CpG hypermethylation at the IFNγ promoter, particularly at transcription factor binding sites. Short-term expanded tumor-specific CTLs derived from four cancer patients exhibited a high frequency of cells with CpG hypermethylation at the IFNγ promoter, with reduced levels of IFNγ mRNA and protein, as well as diminished T cell cytotoxicity. Altogether, these observations suggest a strong association between IFNγ promoter CpG hypermethylation with an impaired IFNγ-mediated response in human tumor-specific CTLs. Our study, therefore, highlights a potential epigenetic target to inform future immunotherapy strategies.

## Materials and Methods

### Generating Antigen-Specific T Cells

Antigen-specific T cells were generated as previously described (19). Briefly, lymphocytes isolated from peripheral blood of cancer patients were stimulated with either tumor antigen-specific SSX2_41−49_ KV9 peptide (KASEKIFYV) or NY-ESO-1_157−165_ SC9 peptide (SLLMWITQC), cytomegalovirus (CMV)-specific pp65_495−503_ peptide (NVLPMVATV), or influenza-specific CY9 peptide (CTELKLNDY) and cultured in Roswell Park Memorial Institute (RPMI)-1640 supplemented with 10% v/v heat-inactivated human AB serum (National Blood Service, UK), 2 mM of l-glutamine and 1% v/v (500 U/ml) of penicillin streptomycin (Sigma-Aldrich, UK) and recombinant human IL-2 (PeproTech, UK) (H10) at 37°C. After 14 days, antigen-specific T cells were fluorescence-activated cell sorting (FACS) sorted with peptide-MHC class I tetramer [human leukocyte antigen (HLA)-A2 KV9 or HLA-A2 SC9 or HLA-A1 CY9) using BD Aria IIlu (BD Biosciences] and were either clonally expanded or short-term population expanded *in vitro*. The T cells were confirmed for antigen specificity using tetramer staining by flow cytometry, and all functional assays were performed using peptide-specific stimulation. T cells were tested for mycoplasma monthly.

### Generation of Human Leukocyte Antigen-Matched Lymphoblastoid B Cell Line

Isolated peripheral blood from a HLA-A2-matched healthy human donor was treated with Epstein–Barr virus (EBV)-containing solution and grown in RPMI-1640 supplemented with 10% v/v fetal calf serum (Sigma Aldrich), 2 mM of l-glutamine, and 1% v/v (500 U/ml) of penicillin streptomycin (Sigma-Aldrich, UK) at 37°C. The cell culture was then treated with cyclosporin A every 3 days within the first 2 weeks to suppress T cells and to allow dominant growth of B cells. Cells were then FACS sorted for CD19^+^ EBV-transformed B lymphoblastoid using BD Aria IIlu (BD Biosciences) and cultured with the above-mentioned culture media.

### Flow Cytometry Staining

T cells were first stained with LIVE/DEAD® Fixable Aqua Dead Cell Stain Kit (Thermo Fisher, UK) before being stained with conjugated antibodies, with each step incubation for 20 min at 4°C. Antibodies used for surface staining used included the following: PerCP/Cy5.5-CD8 (SK1; BD Biosciences; RRID:AB_2687497), PE/Cy7-CD27 (M-T271; BioLegend; RRID:AB_2562258), BV421-CCR7 (G043H7; BioLegend; RRID:AB_11203894), BV711-CD45RA (HI100; BioLegend; RRID:AB_11218999), and fluorescein isothiocyanate (FITC)-CD28 (CD28.2; BD Biosciences; RRID:AB_396071). All samples were acquired on BD LSR Fortessa (BD Biosciences) flow cytometer and analyzed using FlowJo™ v.10 software (Tree Star Inc.).

### T Cell Receptor Sequencing

Briefly, the TCR was sequenced by isolating mRNA using RNAeasy Mini Kit (Qiagen, Germany) and synthesized cDNA using SMARTer RACE cDNA Amplification kit (Takara), PCR amplified using PCR Advantage Kit (Takara), and run on a 1% agarose gel for PCR band confirmation. The PCR product was then purified and transformed with TOP10 competent cells (Thermo Fisher, UK) and plated on Luria broth (LB) agar media; and colony PCR was performed to amplify product before isolating plasmid DNA using Spin Miniprep Kit (Qiagen, Germany). The resulting purified plasmid DNA was sent for sequencing at 100 nM of concentration.

### Intracellular Cytokine Staining

T cells were treated with Monensin and Brefeldin A (BD Biosciences) prior to co-culture with peptide-stimulated lymphoblastoid B cell line (BCL) or HCT116 for 5 h at 37°C. Cells were then surface stained with PerCP/Cy5.5-CD8 (SK1; BD Biosciences; RRID:AB_2687497) before being fixed with Cytofix/Cytoperm™ (BD Biosciences) and stained with conjugated intracellular antibodies including Alexa Flour488-IFNγ (B27; BD Biosciences; RRID:AB_396827) and APC-TNFα (Mab-11; BioLegend; RRID:AB_315264). Samples were acquired on an Attune Nxt flow cytometer (Thermo Fisher Inc.) and analyzed using FlowJo™ v.10 software (Tree Star Inc.).

### Cytokine Production Assessment

For *in vitro* cytokine production, T cell supernatants were collected after 48 h of co-culture at 37°C as mentioned above and quantified using Bio-Plex Pro™ Human cytokine Assay (BioRad) for IFNγ and TNFα production.

### Carboxyfluorescein Succinimidyl Ester-Based Cytotoxic T Lymphocyte Killing Assay

HLA-A2^+^ HCT116 was initially stained with 0.5 μM of carboxyfluorescein succinimidyl ester (CFSE) (Thermo Fisher, UK) before antigen stimulated at five different concentrations and co-cultured with different antigen-specific T cells at 37°C for 6 h. Cells were then stained with APC-E-Cadherin (67A4, BioLegend; RRID:AB_756069) and 7-AAD (BD Biosciences), and cancer cell death was assessed by the CFSE^+^7-AAD^−^ population (dead and non-proliferative cells) present. Samples were acquired on an Attune Nxt flow cytometer (Thermo Fisher Inc.).

### mRNA Expression Analysis

Simultaneous IFNγ and TNFα protein and mRNA levels in antigen-specific T cells were determined using the PrimeFlow™ RNA assay kit (Thermo Fisher Inc.), performed according to manufacturer's instruction. Initially, each T cell type was treated with 10 μl/ml of ImmunoCult™ Human CD3/CD28 T cell Activator (StemCell Technologies) at different time points for 0, 1, 2, and 3 h before being stained with LIVE/DEAD® Fixable Aqua Dead Cell Stain Kit (Thermo Fisher, UK) for 20 min at 4°C before being stained with PE/Dazzle594-CD8 (BioLegend, SK1, RRID:AB_2566515). Cells were then fixed and permeabilized using PrimeFlow Fixation Buffer and PrimeFlow RNA Permeabilization Buffer with RNAse Inhibitors before staining intracellularly for IFNγ and TNFα as mentioned above. Following this, cells were treated with IFNγ and TNFα mRNA target probe sets and incubated for 30 min at 40°C. Hybridization of the probes was performed using PrimeFlow RNA PreAmp Mix, followed with RNA Amp Mix, at 40°C incubation for 30 min for each step. Labeling of hybridized mRNA was then performed using PrimeFlow RNA label probes, containing fluorophore Alexa Fluor 488, Alexa Fluor 647, and Alexa Fluor 750 and analyzed using flow cytometer. Samples were acquired on an Attune Nxt flow cytometer (Thermo Fisher Inc.).

To validate the contribution of CpG hypermethylation on IFNγ mRNA and protein expression, we first treated each tumor-specific CTL clone with 3 μM of 5-aza-2′-deoxycytidine (Sigma Aldrich, UK) for 3 days, in order to induce overall DNA demethylation. The medium containing 5-aza-2′-deoxycytidine was renewed every 24 h, owing to its poor stability After 3 days, simultaneous IFNγ and TNFα protein and mRNA levels were determined using the PrimeFlow™ RNA assay kit (Thermo Fisher Inc.) as described previously.

### Bisulfite Treatment, PCR, and Sequencing

First, 2 × 10^6^ T cells were treated with protein precipitation solution (Qiagen) before being added with isopropanol and then were spun using microcentrifuge to collect pellets. Pellets were then resuspended using 70% ethanol and spun before being resuspended in nuclease-free water. The extracted gDNA was bisulfite treated using EZ DNA methylation-Gold kit (Zymo Research) according to manufacturer's instruction and performed in previous studies. Briefly, gDNA was treated with CT Conversion Reagent solution before being added with M-binding buffer and spun using spin column. Isolated DNA was treated with M-desulfonation buffer to remove salt and washed two times by spinning. DNA was then eluted using nuclease-free water, and concentration was measured using NanoDrop One spectrophotometer (Thermo Fisher Inc.).

Sets of forward and reverse bisulfite primers were designed for each CpG site of the IFNγ promoter (GenBank accession no. J00219), as in Supplementary Table 2. The list of IFNγ bisulfite primers sets is as follows: for CpG site −295 (set sequences: 5′-TGTTTTTAATTATAAGTAAATGATTAATGTGTTTTG-3′ and 5′-CATTATACCCACCTATACCATTCTAATAAAA-3′), for CpG site −186 (5′-GTGGGGAGGTATAAAAAAATTTTTAG-3′ and 5′-CATTTAAATATTATAATTAAAATTTCCTTTAAACTCC-3′), for CpG site −54 (5′-GGATTTAAGGAGTTTAAAGGAAATTTTAATTAT-3′ and 5′-CCTCCTCTAACTACTAATATTTATACCTAAT-3′), and for CpG sites +122 and +128 (5′-TAGTTAAGTTTTTTGGATTTGATTAGTTTGA-3′ and 5′-AATCCTAACAATAACAACCAAAAAAAC-3′). TNFα promoter analysis was performed as a control, with a pair of bisulfite primers sets used to encompass all six proximal CpG sites within the TNFα promoter (set sequences: 5′-ACCCAACCTTTCCTAAAACCTCAAA-3′ and 5′-GTTGTTTTTAGGGGGGGTTTGTAG-3′). The PCR was performed using Advantage-HF 2 PCR kit (Takara Biotech), as per manufacturer instruction. Briefly, 100 nM of PCR primers was used and mixed with a minimum of 100 ng of bisulfite treatment gDNA, HF-PCR buffer solution, 0.2 mM of dNTPs, and 1 U of Titanium *Taq* DNA Polymerase Mix. The cycling parameters were 94°C for 90 s (1 cycle), 94°C for 15 s, 54°C for 60 s, 68°C for 75 s (35 cycles), and 68°C for 180 s (1 cycle). The PCR product was confirmed using 1% agarose gel at 50 V for 60 min. Once band presence was confirmed, the PCR product was cloned using TOPO TA cloning kit for sequencing (Thermo Fisher Inc.). Briefly, the PCR product was cloned into PCR4-TOPO plasmid and transformed on TOP10 *Escherichia coli*-competent bacteria and grown in LB plate with 100 mM of ampicillin. Grown colonies were selected; and Miniprep was performed to isolate concentrated plasmid using Plasmid Miniprep Kit (Qiagen), sequenced using M13 forward and reverse primers with Big Dye Terminator v3 Chemistry (Applied Biosystems) as per manufacturer instruction, and analyzed on an AB-3730 capillary electrophoresis instrument. Data were then further analyzed for methylation presence using SnapGene software (GSL Biotech LLC).

### Statistical Analysis

All graph generation and statistical analyses were conducted using GraphPad Prism v.7 software. Unless stated otherwise, data are summarized as median ± SEM. All statistical details of experiments can be found on each figure legend and Results sections. All *in vitro* T cell experiments were performed three times for each type of experiment. For comparisons between more than two paired groups, two-way ANOVA with Tukey's multiple comparison test was performed. Correlation analysis was performed using non-parametric Spearman test. All tests were two-sided, and differences were considered as statistically significant as *P* < 0.05.

## Results

### Tumor-Specific CD8^+^ T Cell Clones Have Reduced Interferon Gamma Expression and Impaired Cytotoxicity

**Figure 1:**
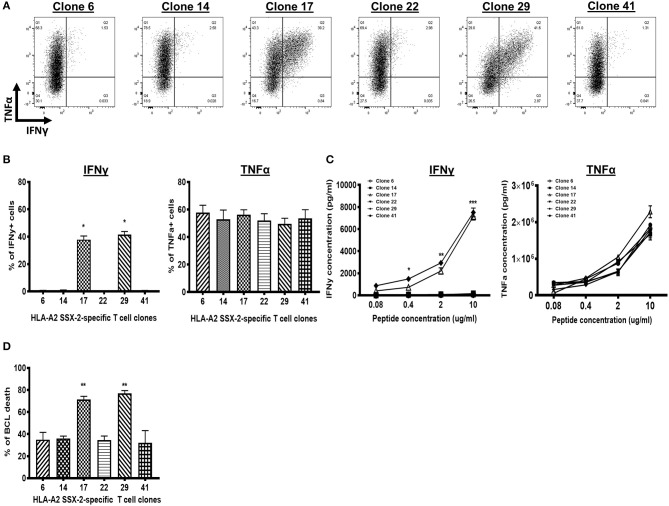
A high frequency of tumor-specific CD8^+^ T cell clones does not express or secrete interferon gamma (IFNγ). **(A)** Flow cytometry dot plots of six synovial sarcoma X (SSX)-2-specific T cell clones. IFNγ is not expressed in four clones (IFNγ^−^ clones: Clones 6, 14, 22, and 41) but is expressed in two clones (IFNγ^+^ clones: Clones 17 and 29). All six clones express TNFα. **(B)** Proportion of IFNγ^+^ cells (left) or TNFα^+^ cells (right) for each T cell clone, after co-culture with peptide-stimulated lymphoblastoid B cell line (BCL). **(C)** Level of IFNγ production (left) or TNFα production (right) by each T cell clone, after co-culture with peptide-stimulated BCL. **(D)** Proportion of BCL cell death following antigen stimulation at 1 μg/ml and co-culture with each T cell clone. *N*, number of experimental repeats = 3. *P < 0.05, **P < 0.01, ***P < 0.001; analysis performed with one-way ANOVA with Tukey's multiple comparison test.

To evaluate IFNγ expression in tumor-specific CTLs, we sorted and clonally expanded HLA-A2-restricted SSX-2-specific CTL clones from a gastric cancer patient, as previously described (19). Upon co-culture with antigen-stimulated HLA-A2-matched BCL, we observed reduced IFNγ expression on four out of six clones (IFNγ^−^ clones: 6, 14, 22, and 41), whereas the other two displayed high IFNγ expression (IFNγ^+^ clones: 17 and 29) (Figure 1A). However, the IFNγ^−^ and IFNγ^+^ clones maintained similarly high expression of TNFα (Figure 1B). We further confirmed that the IFNγ^−^ T cell clones secreted TNFα but not IFNγ, even upon activation with very strong antigen stimulation (Figure 1C; Supplementary Figure 1).

We hypothesized that the IFNγ^−^ T cell clones have altered cytotoxic activity. Indeed, we observed a significantly reduced frequency of BCL death following co-culture with the IFNγ^−^ T cell clones, compared with co-culture with the IFNγ^+^ T cell clones (Figure 1D). This suggests that the IFNγ^−^ T cell clones have reduced cytotoxic response. As each T cell clone has the same TCR and exhibited the same late stage effector memory differentiation phenotype *in vitro* (Supplementary Table 1; Supplementary Figure 2), these data indicate that the reduced cytotoxic response of IFNγ^−^ T cell clones is not due to confounding factors such as different TCR affinity. We infer that the defective IFNγ-mediated responses of the IFNγ^−^ T cell clones underlie their reduced cytotoxic response.

### 5′Methylcytosine-Guanine Hypermethylation of the Interferon Gamma Promoter Contributes to the Interferon Gamma Defect

The CpGs at positions −186 and −54 within the IFNγ promoter, mapped to the transcription factor binding sites, are vital for IFNγ transcription (6, 7). We thus assessed if the lack of IFNγ expression in IFNγ^−^ T cell clones correlates with the methylation of these CpGs at these sites as well as on adjacent CpGs within the promoter (Supplementary Table 2).

**Figure 2:**
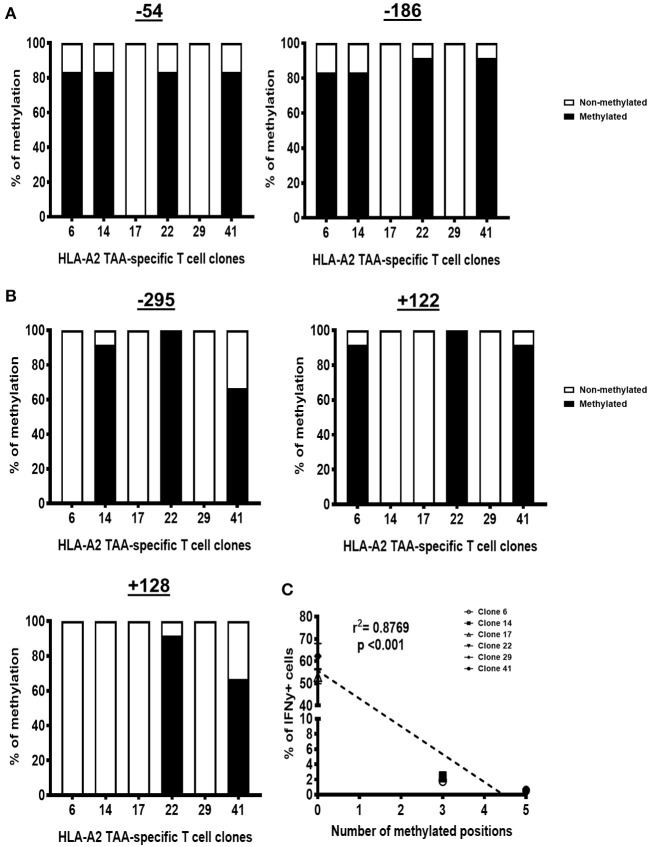
IFNγ^−^ T cell clones have 5′methylcytosine-guanine (CpG) methylation in the interferon gamma (IFNγ) promoter. Proportion of DNA sequences that have CpG methylation at positions −51 and −186 **(A)** as well as at positions −295, +122, and +128 **(B)**. **(C)** The number of methylated sites within the IFNγ promoter correlates with the proportion of IFNγ^−^ cells for each T cell clone. *N*, number of experimental repeats = 3. Analysis performed with non-parametric Spearman correlation test.

To this end, we analyzed the IFNγ promoter of the genomic DNA (gDNA) from each T cell clone by bisulfite sequencing. We found that all the IFNγ^−^ T cell clones were heavily methylated at positions −54 and −186, whereas the IFNγ^+^ T cell clones were unmethylated at these positions (Figure 2A). Adjacent CpGs were also methylated in the IFNγ^−^ clones, including at position −295 (IFNγ^−^ clones: 14, 22, and 41), position +122 (IFNγ^−^ clones: 6, 22, and 41), and position +128 (IFNγ^−^ clones: 22 and 41), whereas all three of these positions are unmethylated in the IFNγ^+^ clones (Figure 2B). The number of methylated CpGs inversely correlated with the frequency of IFNγ^+^ cells (Figure 2C). These data suggest that CpG hypermethylation within the IFNγ promoter can repress the IFNγ expression in tumor-specific CTLs.

In contrast, we did not find any CpG methylation within the TNFα promoter region, specifically at positions +106, +159, +175, +312, +372, and +377 of the proximal part of the promoter (Supplementary Figure 3). This is consistent with the high level of TNFα expression and production by each of these clones.

### The Interferon Gamma Defect Is Associated With Tumor Antigen-Specific Cytotoxic T Lymphocytes

**Figure 3:**
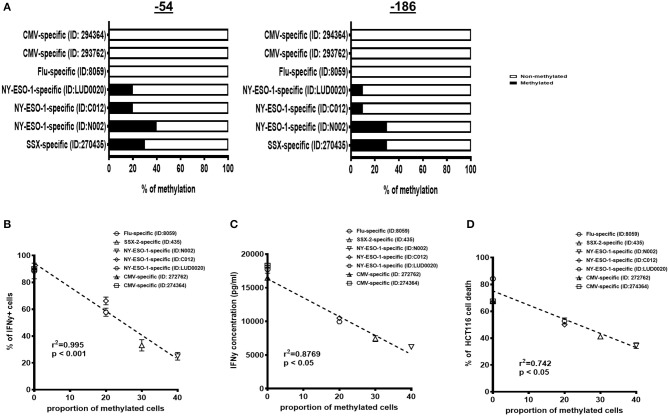
Interferon gamma (IFNγ) methylation and correlation to IFNγ production and T cell cytotoxicity of short-term expanded bulk antigen-specific cytotoxic T lymphocytes (CTLs). **(A)** Proportion of cells with methylation at positions −186 and −54 for each group of antigen-specific T cells [influenza-specific T cells, cytomegalovirus (CMV)-specific T cells, synovial sarcoma X (SSX)-2-specific T cells, and New York-esophageal squamous cell carcinoma-1 (NY-ESO-1)-specific T cells derived from different patients]. The proportion of cells with a hypermethylated IFNγ promoter inversely correlates with the proportion of IFNγ mRNA^+^ cells **(B)** and IFNγ protein production **(C)** expressed for each antigen-specific T cell type. **(D)** The proportion of cells with a hypermethylated IFNγ promoter inversely correlates with HCT116 cancer cell death induced by antigen-specific T cells at 1 μg/ml of antigen stimulation. *N*, number of experimental repeats = 3. Analysis performed with non-parametric Spearman correlation test.

We extended and confirmed these findings in *ex vivo* isolated, short-term expanded, SSX-2-specific or NY-ESO-1-specific CTL lines derived from four different gastric or melanoma cancer patients, as previously described (20, 21) (HLA restriction and antigen specificity as described in Supplementary Table 3). Intriguingly, 10–40% of each short-term tumor-specific CTL lines displayed CpG methylation at positions −186 and −54 within their IFNγ promoter (Figure 3A).

To determine if the CpG methylation was unique to tumor-specific CTLs, we then performed the same analysis with *ex vivo*-isolated and short-term expanded HLA-A2-restricted influenza-specific and CMV-specific CTL lines derived from three different cancer patients. Strikingly, the CpGs at positions −186 and −54 were completely unmethylated in the short-term influenza-specific and CMV-specific CTL lines (Figure 3A). Taken altogether, these observations suggest that a sizeable portion of tumor-specific CTLs could acquire CpG hypermethylation of their IFNγ promoter.

In addition, the frequency of IFNγ promoter-methylated CTL lines was inversely correlated with activation-induced IFNγ expression (Figure 3B) and production (Figure 3C). Most importantly, the cytotoxic killing following co-culture with antigen-stimulated HLA-A2-matched HCT116 cancer cells was also negatively affected (Figure 3D). Altogether, these observations suggest that CpG hypermethylation contributes to the lack of IFNγ synthesis.

### 5′Methylcytosine-Guanine Hypermethylation Is Associated With Reduced Induction of Interferon Gamma Transcription and Protein

**Figure 4:**
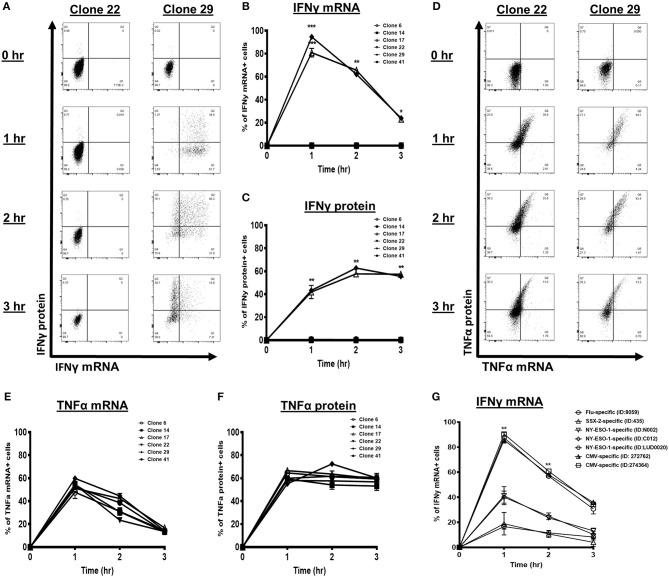
Interferon gamma (IFNγ) kinetics of synovial sarcoma X (SSX)-2-specific T cell clones and short-term expanded bulk antigen-specific cytotoxic T lymphocytes (CTLs). **(A)** Flow cytometry dot plots of IFNγ mRNA and protein levels following co-culture with CD3/CD28 activator antibody at the indicated time points. Proportion of cells with IFNγ mRNA **(B)** and protein **(C)** following co-culture with CD3/CD28 activator antibody at the indicated time points. **(D)** Histogram plots of TNFα mRNA and protein levels following co-culture with CD3/CD28 activator antibody at the indicated time points. Proportion of cells with TNFα mRNA **(E)** and protein **(F)** following co-culture with CD3/CD28 activator antibody at the indicated time points. **(G)** IFNγ mRNA level of each antigen-specific T cell type [influenza-specific T cells, cytomegalovirus (CMV)-specific T cells, SSX-2-specific T cells, and New York-esophageal squamous cell carcinoma-1 (NY-ESO-1)-specific T cells derived from different patients] following treatment with CD3/CD28 activator antibody for 0, 1, 2, and 3 h. *N*, number of experimental repeats = 3. *P < 0.05, **P < 0.01, ***P < 0.001; analysis performed with two-way ANOVA with Tukey's multiple comparison.

Transcription factor accessibility to the binding sites within the promoter is vital for IFNγ transcription (6, 7). To assess the kinetics of IFNγ transcript and protein accumulation in response to T cell activation, we performed a PrimeFlow RNA assay followed by flow cytometry analysis on the SSX-2-specific CTL clones. IFNγ^+^ clones, such as Clone 29, showed a transient increase in IFNγ mRNA levels that peaked at 1 h post activation that then declined (Figures 4A,B). The IFNγ protein levels simultaneously peaked and stabilized 2 h post activation for the IFNγ^+^ T cell clones (Figures 4A,C). In contrast, the IFNγ^−^ clones, such as Clone 22, lacked any IFNγ transcript or protein expression, even at 3 h post activation (Figures 4A–C). These data suggest that the CpG hypermethylation, characteristic of these IFNγ^−^ clones, could impair IFNγ transcription and, in turn, their protein expression.

To validate that the deficiency in IFNγ synthesis driven largely by the CpG hypermethylation of the IFNγ promoter and not a general T cell defect, we used the same approach to assess the TNFα kinetics for each tumor-specific CTL clone. All the IFNγ^+^ and IFNγ^−^ CTL clones showed strong and simultaneous induction of TNFα mRNA and protein, starting at 1 h post activation (Figures 4D–F). Importantly, all CTL clones maintained high levels of TNFα protein at 3 h post activation (Figure 4F). Therefore, the IFNγ^−^ CTL clones likely have intact processing of TNFα, suggesting that the IFNγ defect is specific for IFNγ regulation.

To further confirm that the IFNγ defect is associated with tumor-specific CTLs, we assessed and compared the IFNγ transcription kinetics in the *ex vivo* isolated short-term expanded tumor-specific and virus-specific CTL lines. Compared with the influenza-specific and CMV-specific CTLs, the tumor-specific CTLs showed significantly reduced induction of IFNγ mRNA expression throughout the time course (Figure 4G). The IFNγ transcript peaked at 1 h post activation and then declined for all the CTLs (Figure 4G), therefore indicating that cytokine induction is diminished, not delayed, in tumor-specific CTLs.

### Interferon Gamma Defect Can Be Recovered by DNA Demethylating Treatment

**Figure 5:**
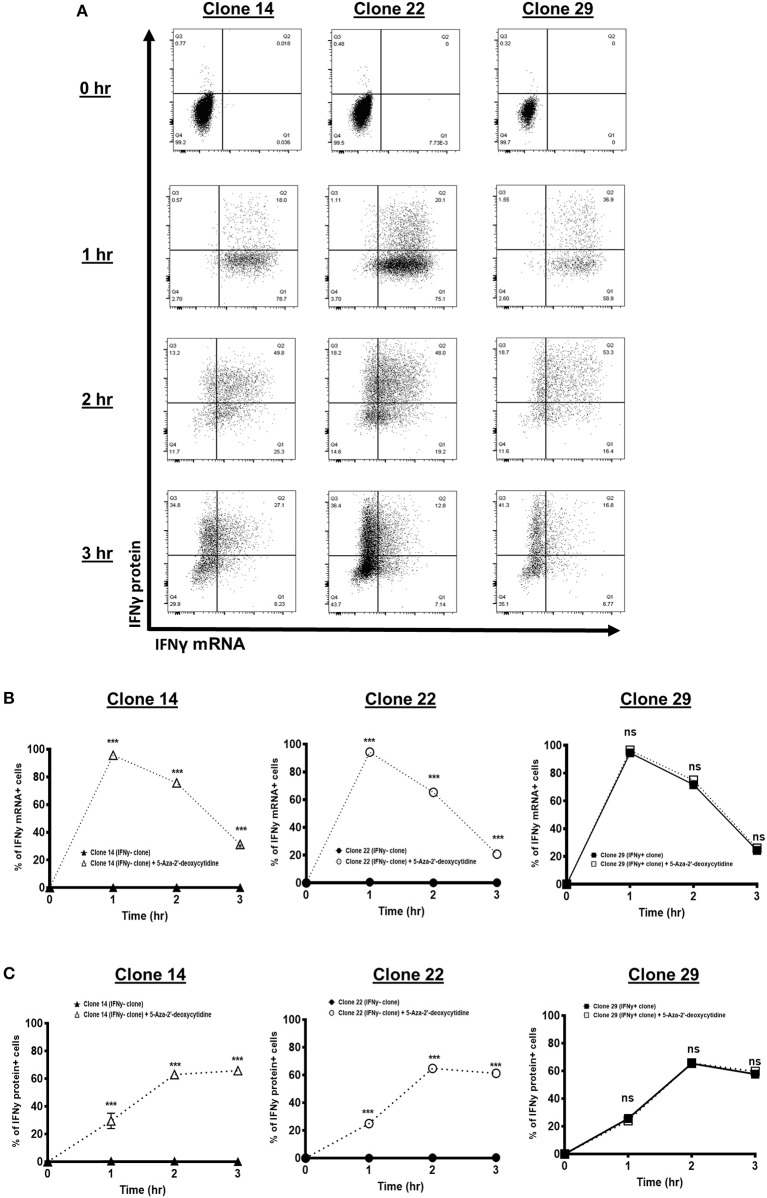
Interferon gamma (IFNγ) kinetics of synovial sarcoma X (SSX)-2-specific T cell clones following DNA demethylating agent treatment. **(A)** Flow cytometry dot plots of IFNγ mRNA and protein expression of two IFNγ^−^ clones (Clones 14 and 22) and one IFNγ^+^ clone (Clone 29), following treatment with DNA demethylating agent (5-aza-2′-deoxycytidine) for 3 days prior to activation with CD3/CD28 activator antibody at the indicated time points. Proportion of cells with IFNγ mRNA **(B)** and protein **(C)** of two IFNγ^−^ clones (Clones 14 and 22) and one IFNγ^+^ clone (Clone 29), following 3 days' treatment with or without 5-aza-2′-deoxycytidine prior to co-culture with CD3/CD28 activator antibody at the indicated time points. *N*, number of experimental repeats = 3. Analysis performed with two-way ANOVA with Tukey's multiple comparison.

Based on the observed correlative analyses, we decided to manipulate the cytosine DNA methylation, in order to link the methylation mechanism to the defective IFNγ transcription. Each tumor-specific CTL clone was first treated with 5-aza-2′-deoxycytidine for 3 days to induce DNA demethylation, prior to assessing their IFNγ mRNA and protein levels. As expected, the IFNγ^−^ clones (Clones 14 and 22) treated with the demethylating agent have restored IFNγ mRNA and protein expression to a level similar to that of the IFNγ^+^ clone (Clone 29) (Figures 5A–C). Particularly, Clones 14 and 22 reached maximum mRNA expression 1 h after T cell activation and maximum protein level 2 h after T cell activation, similar to Clone 29. In contrast, the demethylating agent-untreated IFNγ^−^ clones (Clones 14 and 22) still failed to induce IFNγ expression (Figures 5B,C). This, therefore, indicates that the CpG hypermethylation of these IFNγ^−^ clones acts as the contributing mechanism to the defective IFNγ.

## Discussion

Our work shows that a substantial fraction of tumor-specific CTLs have a compromised IFNγ response and a hypermethylated IFNγ promoter, especially at the CpG positions within the transcription factor binding sites. Importantly, the frequency of cells with a hypermethylated IFNγ promoter correlates with impaired IFNγ induction, IFNγ production, and CTL cytotoxic responses upon T cell activation. This would be predicted to compromise the overall anti-tumor effect of tumor-specific CTLs.

DNA methylation is a key epigenetic regulator of cellular functions. Although cancer cells themselves are known to hyper-methylate their own IFNγ promoter and thus promote tumorigenesis (22), it was not known whether this gene is dysregulated in tumor-specific CTLs, especially at the level of DNA methylation. We have shown, for the first time to our knowledge, that human tumor-specific CTLs can be dysregulated by hypermethylation of the IFNγ promoter, which compromises transcription and, in turn, the protein synthesis of IFNγ. Hypermethylation might be driven by the methyl-CpG binding proteins, such as DNA methyltransferases and enhancer of zester homolog 2 (EZH2), which are known to be upregulated in TILs as well as in cancer cells themselves (23, 24). How a large fraction of tumor-specific CTLs acquire hypermethylation of the IFNγ promoter merits further investigation.

Currently, methods to detect and identify antigen-specific CTLs primarily rely on IFNγ expression and production following stimulation, such as by using ELISpot and ICS (3, 4, 25). Our current study clearly demonstrates that a significant fraction of tumor-specific CTLs have hypermethylation at the IFNγ promoter and IFNγ deficiency. Thus, identifying tumor-specific CTLs solely using IFNγ-based techniques might overlook a large fraction of antigen-specific T cells and could generate unexpected biases in analyses, especially during *ex vivo* evaluation of anti-tumor responses from TILs. Methods to detect multiple cytokines, such as TNFα, should be considered when assessing the presence of tumor-specific TILs and their response in cancer patients.

We observed IFNγ deficiency in tumor-specific CTLs isolated from a small number of melanoma and gastric cancer patients. As our observations clearly provide evidence of IFNγ defects in tumor-specific CTLs, and its negative influence on the cytotoxic and effector responses, further studies should be conducted with larger sample sizes and in different cancer types to decipher the prevalence of IFNγ-deficient tumor-specific CTLs and their relevance to tumor progression and treatment outcome, and the development of novel cancer immunotherapies.

## Data Availability Statement

All datasets generated for this study are included in the article/Supplementary Material.

## Author Contributions

TD conceived the study. TD, YP, and MA designed the study. TD, YP, CL, and MA developed the methodology. CW, TR, and JF acquired the data (e.g., sequencing and cell sorting). MA, XY, SR-M, and YP analyzed and interpreted the data (e.g., statistical analysis, biostatistics, and computational analysis). YP and TD supervised the study. MA, YP, and TD wrote and reviewed the manuscript.

### Conflict of Interest

The authors declare that the research was conducted in the absence of any commercial or financial relationships that could be construed as a potential conflict of interest.
