# Supplementary material for: Defective Interferon Gamma Production by Tumor-Specific CD8+ T Cells Is Associated With 5′Methylcytosine-Guanine Hypermethylation of Interferon Gamma Promoter
Source: Front Immunol. 2020 Mar 5;11:310. doi: 10.3389/fimmu.2020.00310 (PMC7066077; doi:10.3389/fimmu.2020.00310)
Supplement: Supplementary file 1 [file Data_Sheet_1.pdf]

Supplementary Material

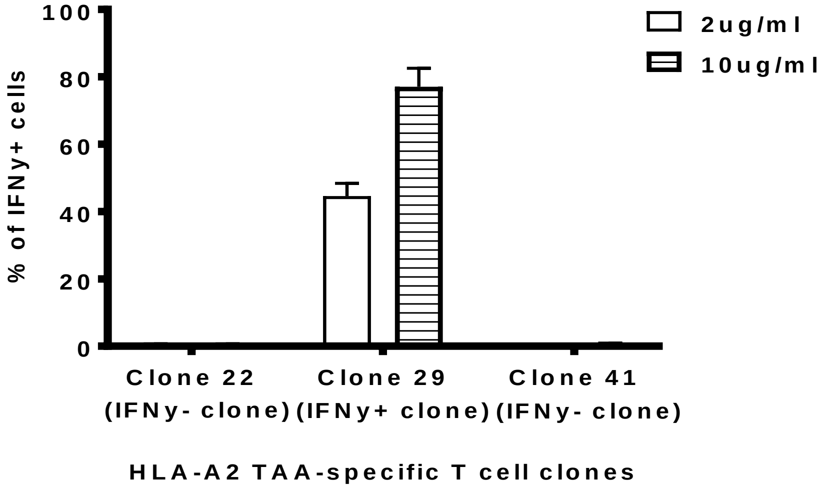

Supplementary Figure 1. Inability of T cell clone to express IFNγ at high antigen-stimulation. T cell clones 22 and 41 do not express IFNγ, even when co-cultured with 10µg/ml peptide-stimulated BCL. Only T cell clone 29 expresses IFNγ, at both 1µg/ml and 10µg/ml peptide-stimulated BCL. N, number of experimental repeats= 3. * represents P-value less than 0.5, analysis performed with one-way ANOVA with Tukey's multiple comparison test.

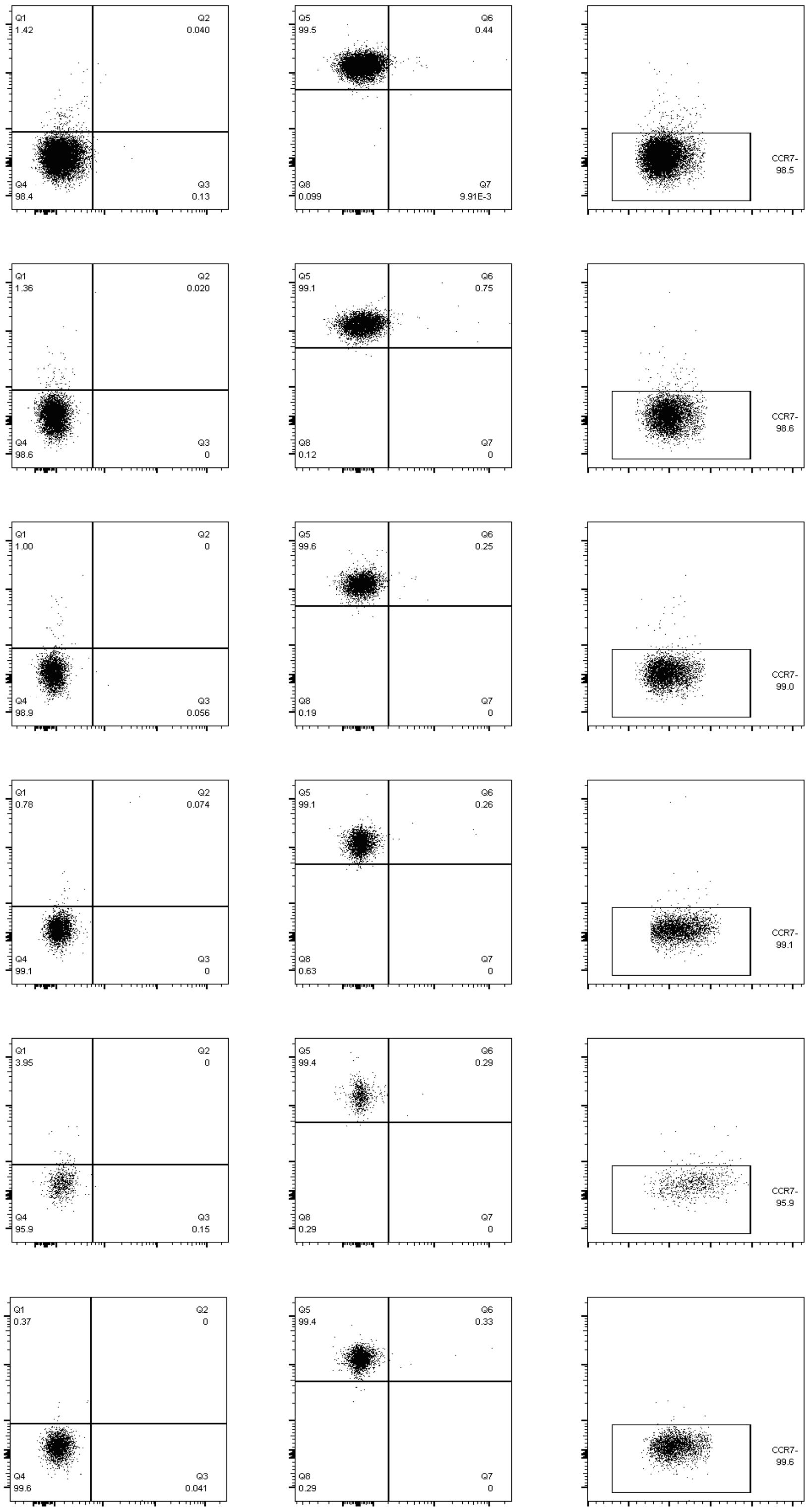

Supplementary Figure 2. Surface phenotype of SSX-2-specific T cell clones. Representative dot plots showing that all six T cell clones have surface expression of CD45RA, CD45RO, CCR7, CD27 and CD28 *in vitro*.

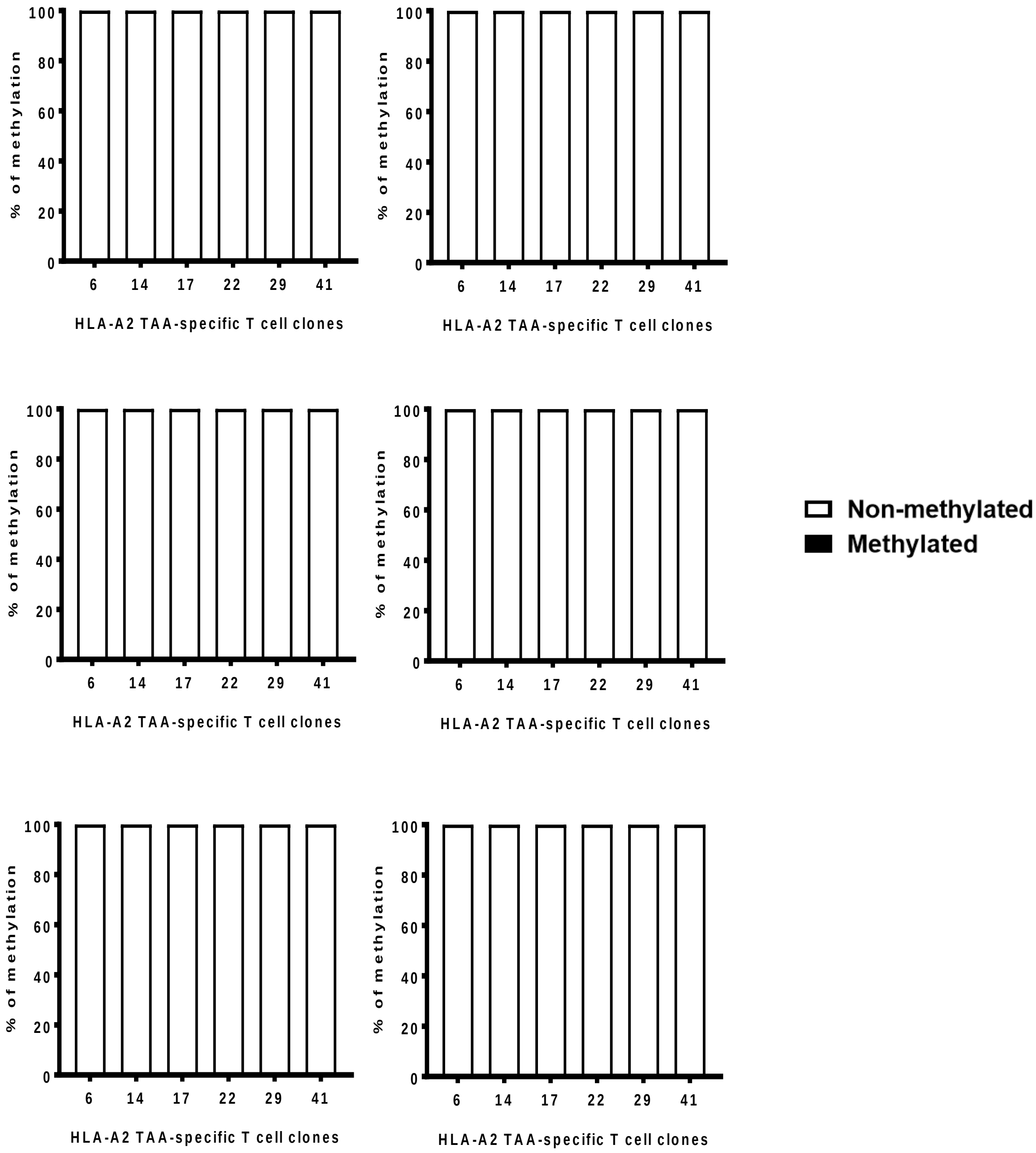

Supplementary Figure 3. CpG methylation of TNFα promoter. In contrast to IFNγ methylation, no presence of CPG methylation was found on each of the tumor-specific CTL clones tested.

Supplementary Table 1. The T cell receptor of the six SSX-2-specific T cell clones.

SSX-2-specific clone ID	TCRα	CDR3 (aa)	TCRβ	CDR3 (aa)
Clone 6	TRAV 8-6 TRAJ 30	CAVSDQNRDDKIIFGKG	TRBV 6-1 TRBJ 2-7	CASSQGLTYGPSSYEQTFGPG
Clone 14	TRAV 8-6 TRAJ 30	CAVSDQNRDDKIIFGKG	TRBV 6-1 TRBJ 2-7	CASSQGLTYGPSSYEQTFGPG
Clone 17	TRAV 8-6 TRAJ 30	CAVSDQNRDDKIIFGKG	TRBV 6-1 TRBJ 2-7	CASSQGLTYGPSSYEQTFGPG
Clone 22	TRAV 8-6 TRAJ 30	CAVSDQNRDDKIIFGKG	TRBV 6-1 TRBJ 2-7	CASSQGLTYGPSSYEQTFGPG
Clone 29	TRAV 8-6 TRAJ 30	CAVSDQNRDDKIIFGKG	TRBV 6-1 TRBJ 2-7	CASSQGLTYGPSSYEQTFGPG
Clone 41	TRAV 8-6 TRAJ 30	CAVSDQNRDDKIIFGKG	TRBV 6-1 TRBJ 2-7	CASSQGLTYGPSSYEQTFGPG

Supplementary Table 2. PCR forward and reverse primers used to identify and sequence IFNγ gene locations containing CpG positions.

CpG position	Location	Gene sequence
1	-295	GGC**CG**AAG
2	-186	CAT**CG**TCA
3	-54	ATA**CG**TAA
4	+122	TCT**CG**GAA
5	+128	AAA**CG**ATG
6	+171	CAT**CG**TTT

Reside in transcription factor binding site (arrows pointing to CpG positions 2 and 3)

Supplementary Table 3. HLA-restriction and antigen-restriction by each antigen-specific T cells from different cancer patients

Patient ID	T cells' HLA-restriction	Antigen specificity
8059	HLA-A2	Influenza-specific CY9 peptide (CTELKLNDY)
435	HLA-A2	$SSX2_{41-49}$ KV9 peptide (KASEKIFYV
N002	HLA-A2	NY-ESO-$1_{157-165}$ SC9 peptide (SLLMWITQC)
C012	HLA-A2	NY-ESO-$1_{157-165}$ SC9 peptide (SLLMWITQC)
LUD0020	HLA-A2	NY-ESO-$1_{157-165}$ SC9 peptide (SLLMWITQC)
272762	HLA-A2	CMV-specific $pp65_{495-503}$ peptide (NVLPMVATV)
274364	HLA-A2	CMV-specific $pp65_{495-503}$ peptide (NVLPMVATV)
